# Supplementary material for: Toxic Metals and Metalloids in Infant Formulas Marketed in Brazil, and Child Health Risks According to the Target Hazard Quotients and Target Cancer Risk
Source: Int J Environ Res Public Health. 2022 Sep 6;19(18):11178. doi: 10.3390/ijerph191811178 (PMC9517614; doi:10.3390/ijerph191811178)
Supplement: Supplementary file 1 [file ijerph-19-11178-s001.zip › ijerph-1876799-supplementary.pdf]

Supplementary file S1

Table S1. Codes for the commercial infant formulas evaluated in the present study.

Phase 1 code (0-6 months)	Batch code	Phase 2 code (6-12 months)	Batch code
ME1	ME1A<br>ME1B<br>ME1C	**ME2**	ME2A<br>ME2B<br>ME2C
NC1	NC1A<br>NC1B<br>NC1C	**NC2**	NC2A<br>NC2B<br>NC2C
NN1	NN1A<br>NN1B<br>NN1C	**NN2**	NN2A<br>NN2B<br>NN2C
DM1	DM1A<br>DM1B<br>DM1C	**DM2**	DM2A<br>DM2B<br>DM2C
DA1	DA1A<br>DA1B<br>DA1C	**DA2**	DA2A<br>DA2B<br>DA2C

Five phase 1 and five phase 2 distinct formulas were selected, comprising three distinct batches of each brand totaling thirty samples (N = 30). Samples were coded, where brands are represented by two capital letters, followed by the recommended phase (1 or 2) and batches of each brand are identified using capital letters (A, B or C).

Supplementary file S2

Table S2. Parameters and variables used in the calculation of THQ and TCR.

IFs	n	EF (day)	ED (year)	IFRI ($g \cdot day^{-1}$)	WAB (kg)	TA (day)
**Phase 1 infant formulas**						
ME1	3	182.5	0.5	117.5	6.1	91.25
NC1	3	182.5	0.5	123.5	6.1	91.25
NN1	3	182.5	0.5	125.1	6.1	91.25
DM1	3	182.5	0.5	111.9	6.1	91.25
DA1	3	182.5	0.5	117.5	6.1	91.25
**Phase 2 infant formulas**						
ME2	3	182.5	0.5	137	9.1	91.25
NC2	3	182.5	0.5	131.6	9.1	91.25
NN2	3	182.5	0.5	98.7	9.1	91.25
DM2	3	182.5	0.5	128	9.1	91.25
DA2	3	182.5	0.5	137	9.1	91.25

THQ, target hazard quotient; TCR, target cancer risk; Ifs, infant formulas; EF, exposure frequency; ED, exposure duration; IFRI, infant formula rate ingestion; WAB, average body weight; TA, average exposure time (EF x ED).

Supplementary file S3

**Table S3.** Sub-chronic oral RfDo ($mg \cdot kg^{-1}$) of metals and metalloids		
**Elements**	**RfDo**	**Reference**
Al	1.0	[41]
As (iAs)*	0.0003*	[18]
Cd	0.001 (food)	[18]
Sn	0.6	[42]
Hg (MeHg)*	0.0001	[18]
Pb	0.0035	[18]
U	0.003	[18]

*The reference doses for As are based on the inorganic form (iAs) and for Hg are based on methyl mercury (MeHg). ATSDR, Agency for Toxic Substances and Disease Registry [41, 42]; U.S. EPA, United States Environmental Protection Agency [18]. Sub-chronic oral RfDs were specifically established to be a protective threshold for elements following a short-term exposure.

Supplementary file S4

Table S4. Limit of detection (LOD) and limit of quantification (LOQ), in $mg \cdot kg^{-1}$, for each element determined in infant formulas samples.

Toxic Elements	LOD	LOQ
Hg	0.003	0.011
As	0.003	0.011
Cd	0.001	0.002
Pb	0.003	0.010
Al	0.129	0.430
Sn	0.002	0.005
U	0.0004	0.001
